# Supplementary material for: Transport and excitations in a negative-U quantum dot at the LaAlO3/SrTiO3 interface
Source: Nat Commun. 2017 Aug 30;8:395. doi: 10.1038/s41467-017-00495-7 (PMC5577219; doi:10.1038/s41467-017-00495-7)
Supplement: Supplementary file 1 — Supplementary Information [file 41467_2017_495_MOESM1_ESM.pdf]

Description of Supplementary Files

File Name: Supplementary Information
Description: Supplementary Figures, Supplementary Notes and Supplementary References

File Name: Peer Review File

Supplementary Note 1. CHARACTERIZATION OF THE SUPERCONDUCTING LEADS

Current-voltage characteristics are measured to find the critical temperature T_{c} and critical magnetic field B_{c}^{z} of the source and drain leads. A current is applied using a 10 MΩ bias resistor, and the voltages V_{L}, V_{R} are measured across the side leads of the device, as indicated in Fig. 1 of the manuscript.

Supplementary Fig. 1 shows the current-voltage characteristics for varying temperatures, and 2 for varying perpendicular magnetic field. The red curve indicates a current sweep from a negative to a positive value, while the blue curve indicates a sweep in the opposite direction. At the critical current I_{c} the device switches from a superconducting to a resistive state. I_{c} is extracted from the data using a threshold detection. The data is fitted to the Ambegaokar-Baratoff relation, based on two weak links with different parameters for T_{c} and R_{JJ}. More details on the hysteretic nature of the IV curve and the theory fit to $I_{\mathrm{c}}(T)$ can be found in [1].

We find a critical temperature $T_{\mathrm{c}} \approx 270$ mK and out-of-plane critical magnetic field $B_{\mathrm{c}}^{z} \approx 90$ mT, similar to previous studies of the room-temperature grown LAO/STO samples [1]. From measurements of samples grown in similar growth conditions and sheet resistance, we estimate the in-plane critical magnetic field $B_{c}^{y} \approx 2\,\mathrm{T}$.

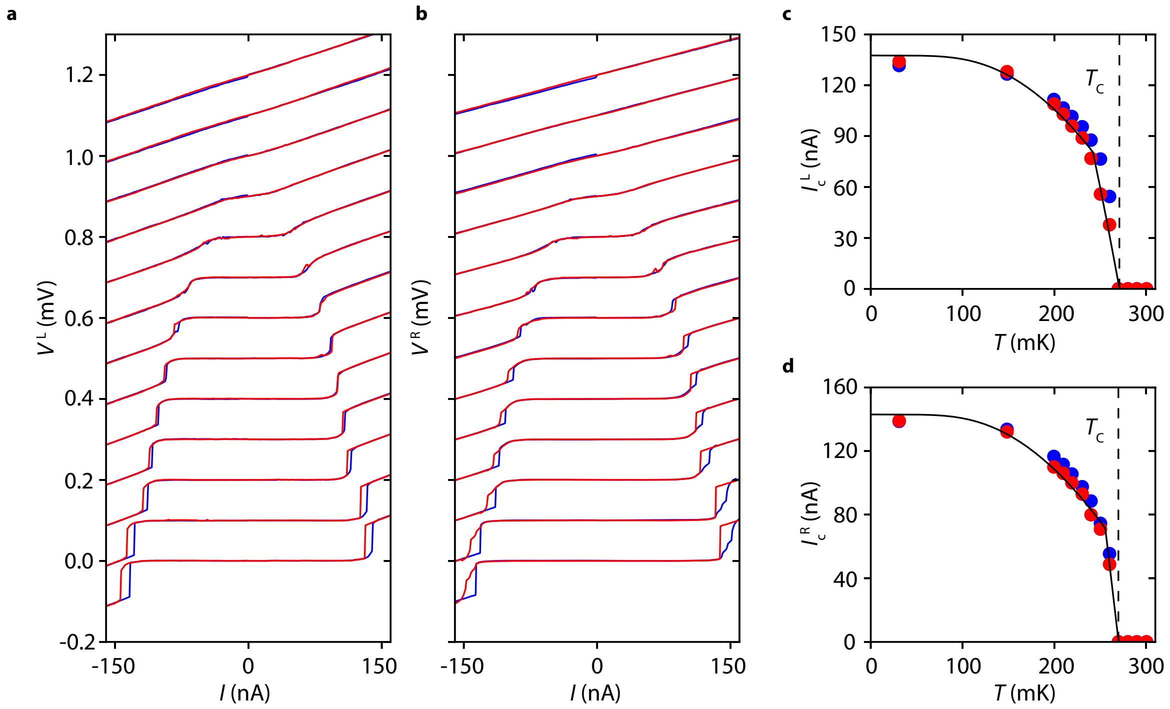

Supplementary figure 1. **Dependence of current-voltage (IV) characteristics of the leads on temperature.** IV-characteristics of the leads **a** to the left and **b** right of the dot (L,R) as a function of temperature, where red (blue) curves indicate sweeping the current in the positive (negative) direction. Extracted critical current **c** I_{c}^{L} and **d** I_{c}^{R}, indicated by red (blue) markers for the positive (negative) sweep direction.

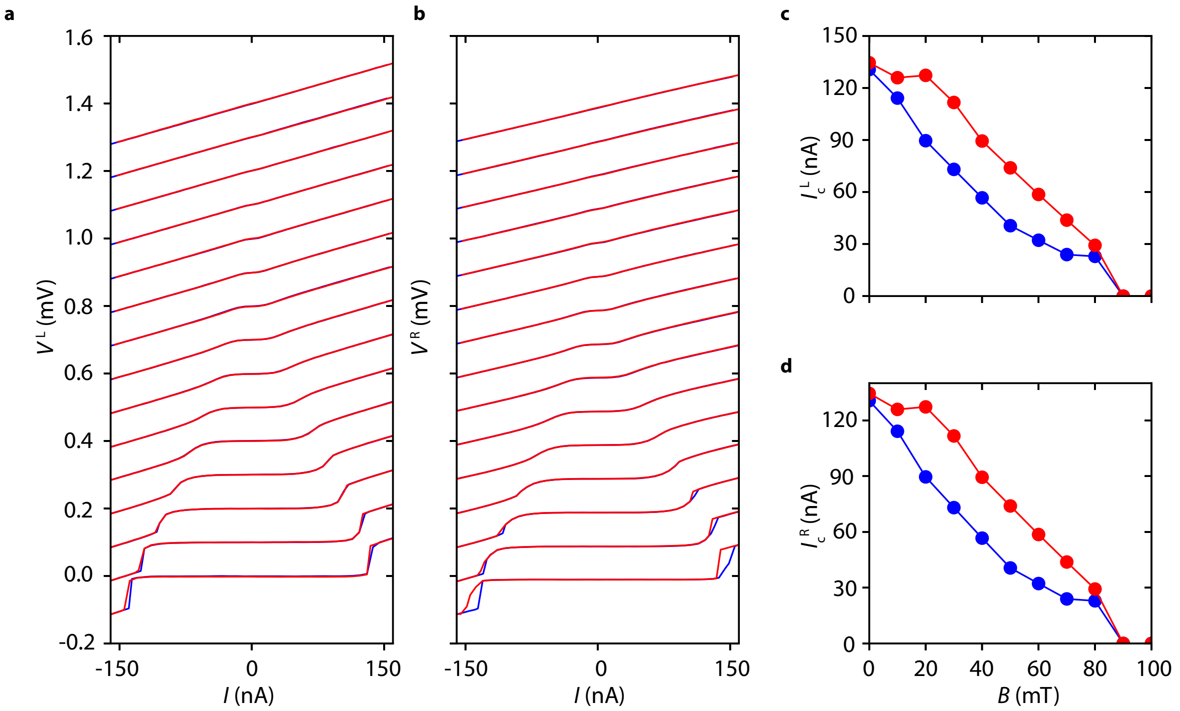

Supplementary figure 2. **Dependence of current-voltage (IV) characteristics of the leads on magnetic field.** IV-characteristics of the leads **a** to the left and **b** right of the dot (L,R) as a function of magnetic field, where red (blue) curves indicate sweeping the current in the positive (negative) direction. Extracted critical current **c** I_c^L and **d** I_c^R, indicated by red (blue) markers for the positive (negative) sweep direction.

Supplementary Note 2. BIAS SPECTROSCOPY

Figures 3 and 4 show the full-range bias spectroscopy for varying external magnetic fields for V_g <-0.6 V. We find a negative charging energy $U \approx -160\mu$eV (white arrows) and diamond height $\Delta E \approx 600$ μeV (red and orange arrows in Supplementary Fig. 3) which is associated with the level spacing ΔE and charging energy E_C. Because of our split-gate device geometry, as dot occupation is increased, simultaneously the tunnel barriers are lowered and tunnel coupling between the dot and the reservoirs is increased. This becomes evident from the closing of the transport gap around zero bias as gate voltage is increased, shown in Supplementary Fig. 4.

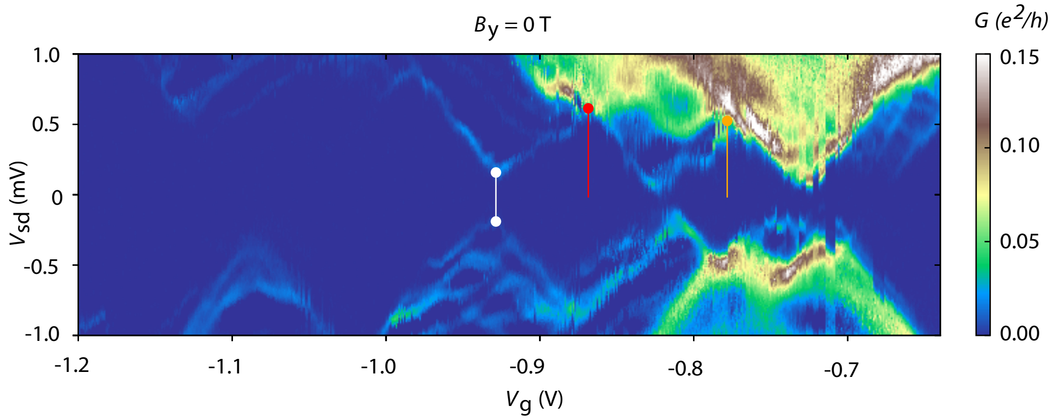

Supplementary figure 3. **Bias spectroscopy of the weak coupling regime at B_y = 0 T.** The height of two subsequent diamonds is indicated by red and orange markers and lines. The size of the transport gap $2|U|$ is indicated by a white line.

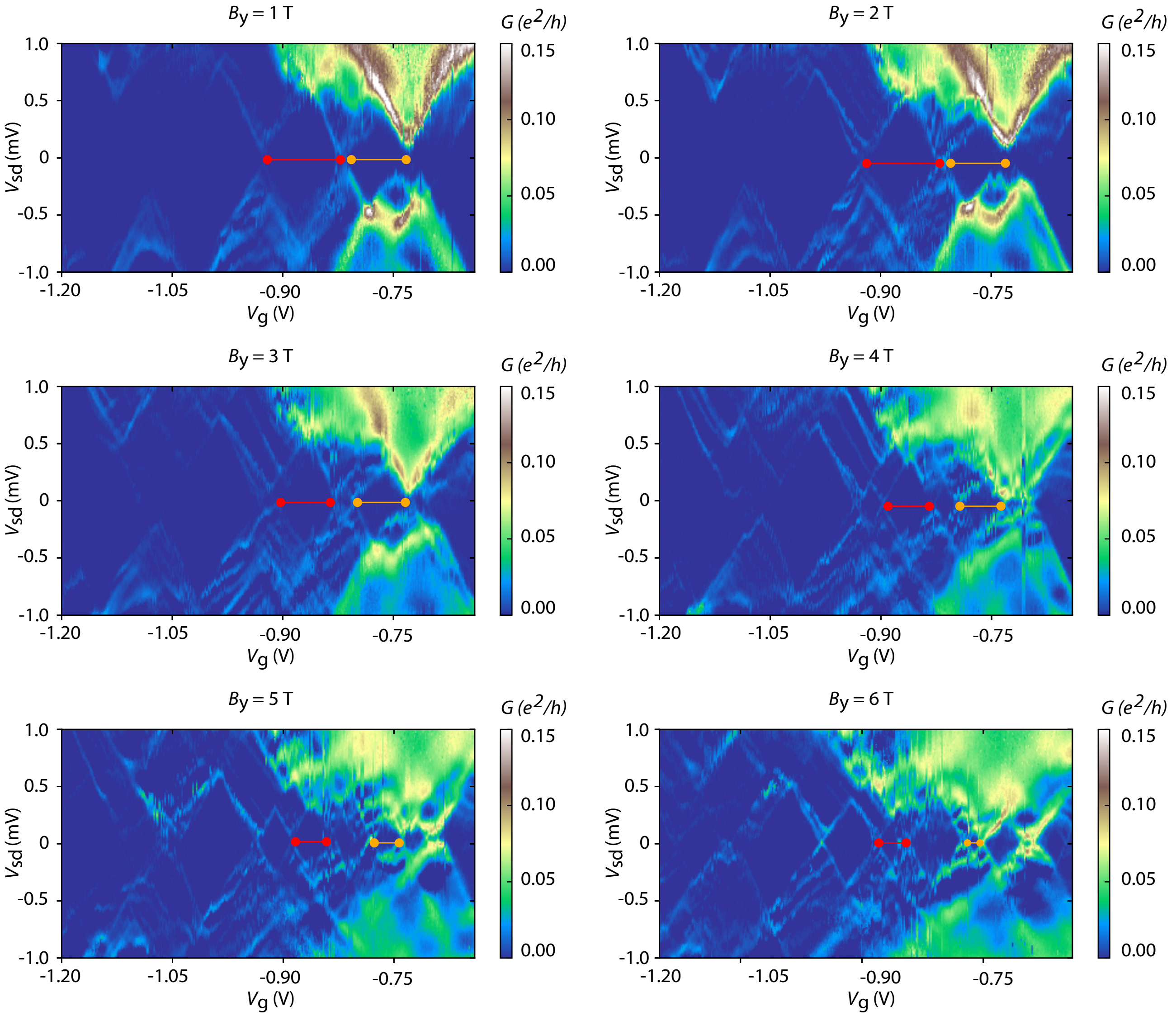

Supplementary figure 4. **Bias spectroscopy of the weak coupling regime at $B_y = 1$ to 6 T.** The width of two diamonds of even occupation is indicated by red and orange markers and lines.

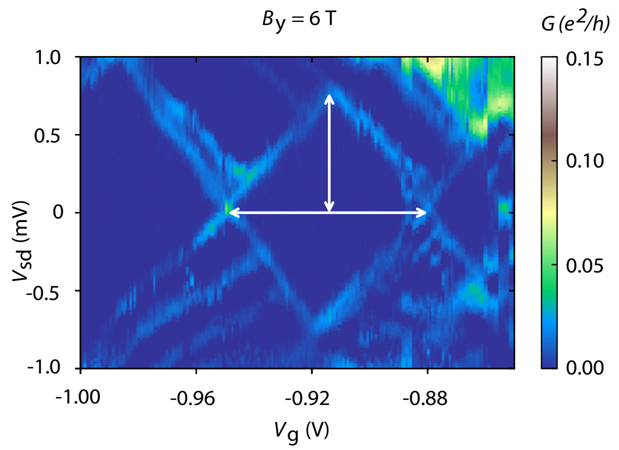

Supplementary figure 5. **A zoom-in of the bias spectroscopy at $B_y = 6\,\mathrm{T}$.** Arrows indicate the diamond height and width used to calculate α_g.

In Supplementary Fig. 5 a zoom-in is shown for the first odd diamond at B = 6 T. The lever arm α_g of the top gates is extracted from the height and width of the diamond, which are indicated in the figure by arrows, with the gate coupling coefficient $\alpha = C_g/C_\Sigma = \frac{\Delta V_{sd}}{\Delta V_g} \approx 0.005$.

Measurements in the x and z-directions revealed that $B_p^{x,y} > 1,\;$ T, beyond our current range for the 6,1,1 vector magnet. This shows that even though the critical fields of the superconducting state of the leads are much lower (by a factor ~ 10) in the perpendicular direction, no such strong reduction occurs for B_p (the details of the measurements will be reported elsewhere). Importantly, we have in general found no measurable consequences of superconductivity in the weak coupling regime supporting the use of normal metal reservoirs in the rate-equation model used to calculate the bias-spectroscopy in the weak coupling regime (Fig. 2 of main manuscript).

Supplementary Note 3. MAGNETIC FIELD DEPENDENCE OF CONDUCTANCE PEAKS

The magnetic field dependence of the conductance peaks is measured for the three axis directions B_{x}, B_{y} and B_{z} using a 6-1-1 vector magnet. Supplementary figure 6 shows the full range for the B_{y} dependence from which the conductance peak at V_{g} = -0.92 V was studied in the manuscript. The B_{x} and B_{z} dependencies did not reveal any splitting of the conductance peaks.

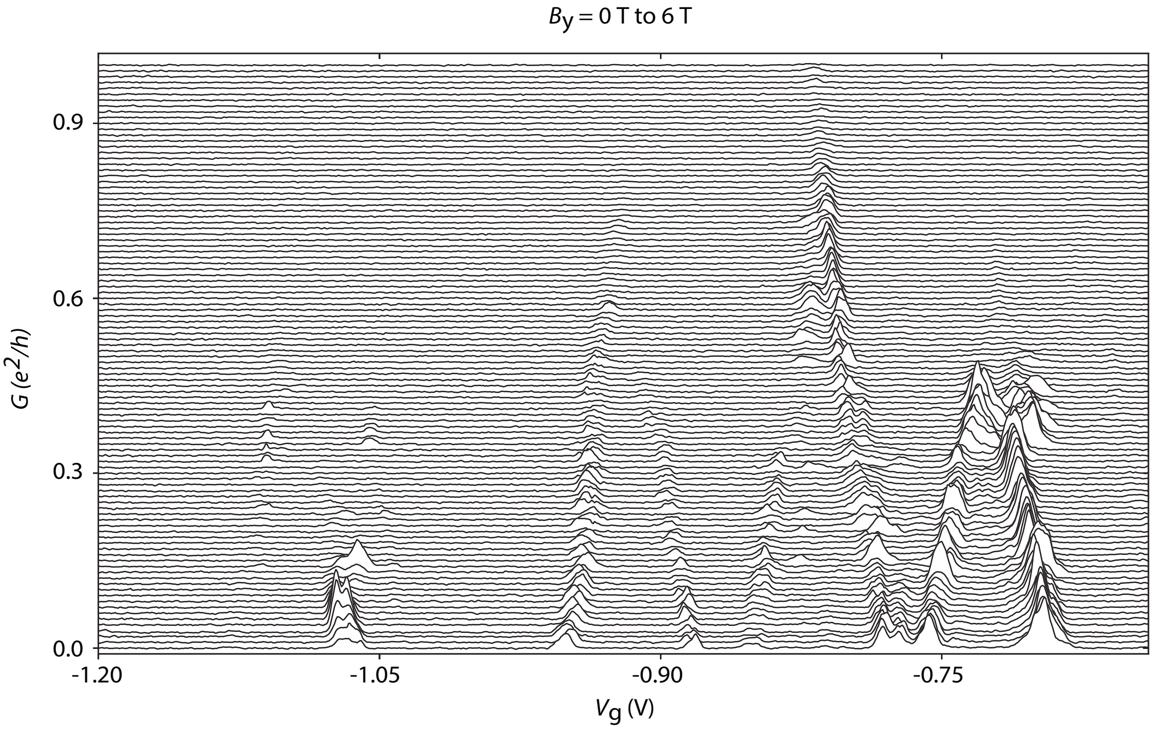

Supplementary figure 6. **The magnetic field dependence of the conductance peaks at zero bias voltage V_{sd} for varying magnetic field.** Each trace is taken at a finite magnetic field B_{y} from 0 to 6 T and offset vertically.

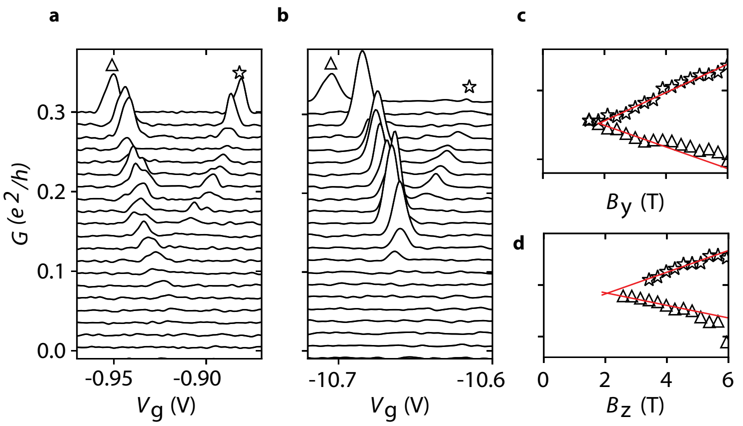

Supplementary figure 7. **Magnetic field dependence of the conductance vs. gate voltage in subsequent cool downs.** **a,b** The dependence of the first conductance peak on magnetic field in the y and z-direction, measured in two subsequent cool downs of the same device. Each trace is taken at a finite magnetic field B_y from 0 to 6 T and offset vertically. **c,d** The magnetic field dependence of the peak positions, each peak indicated by $\triangle$, $\star$, respectively, as indicated in panels (a, b). The points are then fit to two linear functions, indicated by red lines, which intersect at the pair-splitting field B_p^y and B_p^z. A more detailed fit is shown in Supplementary figure 9

In a subsequent cool down, the sample was rotated such that B_x could be increased up to 6 T. The magnetic field dependence of the conductance peak at V_g = -.92 V and extracted peak positions are shown in Supplementary Fig. 7. The pairing field at which the peaks split up is found using linear fitting of the data above B = 2 T. Regions in which the peaks strongly deviate from linearity in V_g due to gate switching have been omitted from the fitting range. The values of the pairing field at which the linear fits cross are found to be $B_\mathrm{p}^\mathrm{y} = 1.8\,\mathrm{T}$ and $B_\mathrm{p}^\mathrm{z} = 2.06\,\mathrm{T}$. It seems that in the closed regime of the dot, no strong angle dependence on magnetic field is present. However, the thermal cycle may have altered the disorder potential and important parameters of the sample that may alter U and/or g.

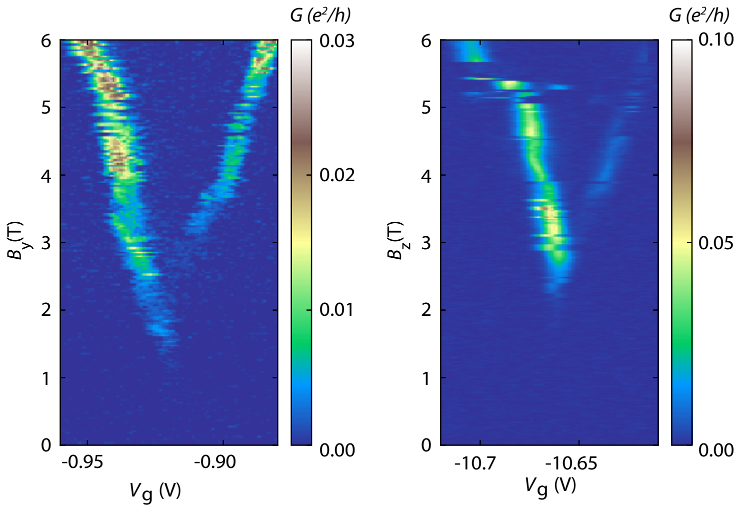

Supplementary figure 8. **Magnetic field dependence of the conductance vs. gate voltage in subsequent cool downs.** A heat map representation of the data presented in Supplementary figure 7 (a, b). The color scale indicates the measured conductance with sweeping the gate voltage V_g on the x-axis and magnetic field B_y (left panel) and B_z (right panel) on the y-axis.

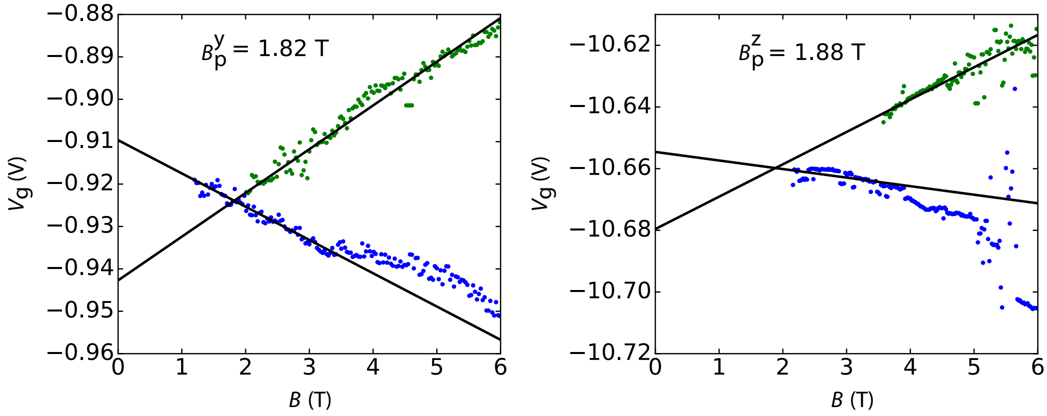

Supplementary figure 9. **Magnetic field dependence of the conductance vs. gate voltage in subsequent cool downs.** The dependence $G(V_g)$ is fitted to a Gaussian, from which the gate voltage V_g at the center of the conductance peaks in V is extracted with dependence on B(T). Here, these values are indicated by green and blue dots. The points are then fit to two linear functions, indicated by black lines, which intersect at the pair-splitting field B_p^y (left panel) and B_p^z (right panel).

Supplementary Note 4. NEGATIVE U MODEL

All transport calculations shown in this work are based on the single-orbital Anderson model. This is in a sense the simplest possible model which can exhibit the desired physics and has only a single orbital which can be occupied with one electron of each spin projection, and with a double occupation associated with an additional charging energy U. This model has been used in many theoretical studies of electron transport in quantum dots, and the only difference here is that we take $U < 0$. The Hamiltonian of the quantum dot coupled to the leads is given by

$$H = H_{QD} + \sum_{r=s,d} H_r + \sum_{r=s,d} H_{Tr}. \tag{1}$$

Here

$$H_{QD} = \sum_{\sigma} \varepsilon_\sigma n_\sigma + U n_\uparrow n_\downarrow \tag{2}$$

describes the quantum dot (QD), which has a single orbital that can be occupied by electrons with spin projection $\sigma = \uparrow, \downarrow$ with associated occupation number $n_\sigma = d_\sigma^\dagger d_\sigma$ and energy ε_σ, where $\varepsilon_\uparrow - \varepsilon_\downarrow = g\mu_B B$. U is the additional energy cost associated with double occupation of the dot orbital, which becomes negative in the case studied here. The source ($r = s$) and drain ($r = d$) are described by noninteracting electrons

$$H_r = \sum_{k\sigma} \varepsilon_{k\sigma r} n_{k\sigma r}, \tag{3}$$

with $n_{k\sigma r} = c_{k\sigma r}^\dagger c_{k\sigma r}$. We assume that those states are always occupied according to a Fermi distribution, meaning that the source and drain are assumed to remain individually in equilibrium (at different chemical potentials) also when a source-drain voltage is applied. Finally, tunneling of electrons between the source/drain and the QD is described by the tunneling Hamiltonian

$$H_{Tr} = \sum_{k\sigma r} t_{k\sigma r} c_{k\sigma r}^\dagger d_\sigma + H.c. \tag{4}$$

For the transport calculations we use the real-time diagrammatic technique [2] to perform a perturbation expansion to order $H_{Tr}^4 \sim \Gamma^2$ for the reduced density matrix of the QD, as well as the current through the dot [3, 4]. This theory takes into account all coherent one- and two-electron tunneling processes, including, for example, single-electron tunneling and cotunneling, as well as the electron pair tunneling terms investigated in [5]. We note that this transport theory takes the interaction U into account in a non-perturbative manner, which is crucial to capture the experimentally observed transport features.

As described in the manuscript, the experiment is compared to transport calculations [3, 4]. Bias spectroscopy for varying magnetic field is calculated and shown in Supplementary figure 10 for negative U, for negative U and asymmetric tunnel coupling and for positive U. In the case of negative U, before a magnetic field is applied, the (0 $\rightarrow$ 1) transition is not allowed as accessing it requires a finite energy. This can either be achieved with a bias voltage $V_{\mathrm{sd}} > U$ or a magnetic field such that $E_{\mathrm{Z}} > U$.

For asymmetric tunnel barriers, excited state tunneling originating from one lead is favored. This is evident from the simulations in Supplementary Fig. 10, indicated by "$U < 0$, asymmetric tunnel barriers" where $\Gamma_{\mathrm{L}} = 10 \times \Gamma_{\mathrm{R}}$. In the case of positive U, the $(0 \rightarrow 1)$ transition dominates the $(0 \rightarrow 2)$ transition in terms of transport.

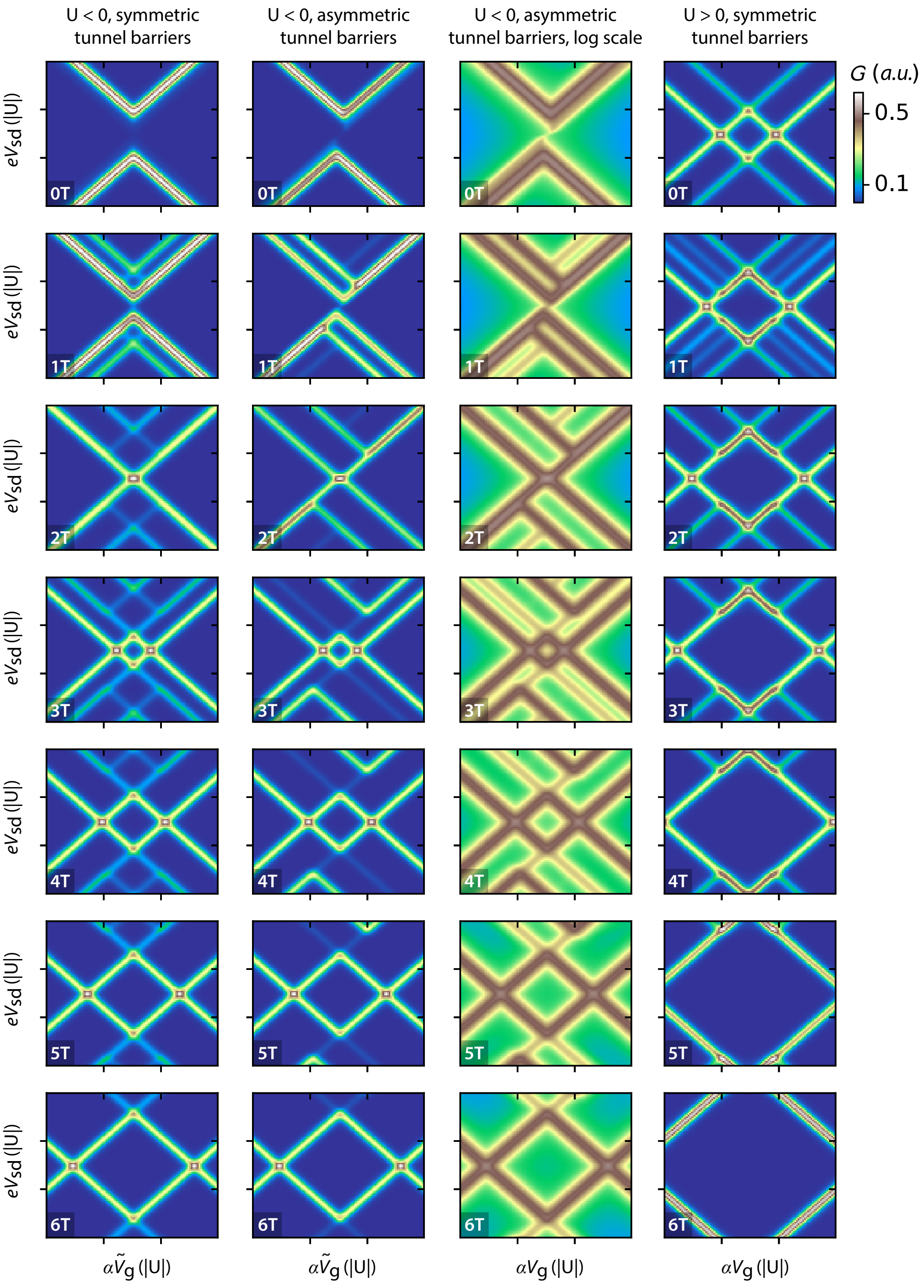

Supplementary figure 10. **Transport calculations for varying magnetic fields and asymmetry.** Different rows shows calculations for different magnetic fields as labeled on each panel. First column shows the results for a QD with $U < 0$ and symmetric tunnel barriers. The results for $U < 0$ and asymmetric tunnel barriers ($\Gamma_{\mathrm{L}} = 10 \times \Gamma_{\mathrm{R}}$) as in the main manuscript is shown in the second and third columns for normal and logarithmic scales, respectively. For comparison, the fourth column shows the results for the conventional quantum dots with $U > 0$ and symmetric barriers.

Supplementary Note 5. TEMPERATURE DEPENDENCE OF CONDUCTANCE PEAKS IN REGIMES OF ZERO AND FINITE MAGNETIC FIELD

To extract the temperature dependence of the peak height, we fit a multiple peak model to the data using the method of least squares. First, a peak finding algorithm is used to find the height and location V_n of each peak n as an initial guess to the fitting procedure. The curves are then fitted to a multiple Gaussian model to provide the initial condition for a least squares fit to the following multiple peak model

$$G = \sum_n \frac{A_n}{\cosh^2[\alpha_g(V_\mathrm{g} - V_\mathrm{n})/k_\mathrm{B}T_\mathrm{el}]} \tag{5}$$

which is expected for a thermally broadened Coulomb Blockade peak in the sequential tunneling regime[6] (i.e. for $B > B_\mathrm{p}$) and also agrees up to fourth order in with the theoretical lineshape $\propto \delta V_\mathrm{g}/\sinh(\delta V_\mathrm{g})$ for pair tunneling[5]. The fit is excellent and the fitted peaks have amplitudes A_n, positions V_n and widths σ_n for the n conductance peaks, $\alpha_\mathrm{g} = 0.0052$ the gate voltage to energy conversion or lever arm. A and full width at half maximum (FWHM) are extracted from calculations and scaled to the data. For the sequential tunneling regime, the width relates to the electron temperature T_el[6] ($3.53k_\mathrm{B}T_\mathrm{el} = \alpha_\mathrm{g}\mathrm{FWHM(V)}$) and for the $B = 0$ case where tunneling consists of both thermally excited sequential tunneling and pair tunneling [5] the FWHM scales linearly with T_el [5]. Upon cooling the fitted FWHM stays constant until it saturates for $T \approx 100$mK, which we attribute to a finite electron temperature in combination with lifetime broadening.

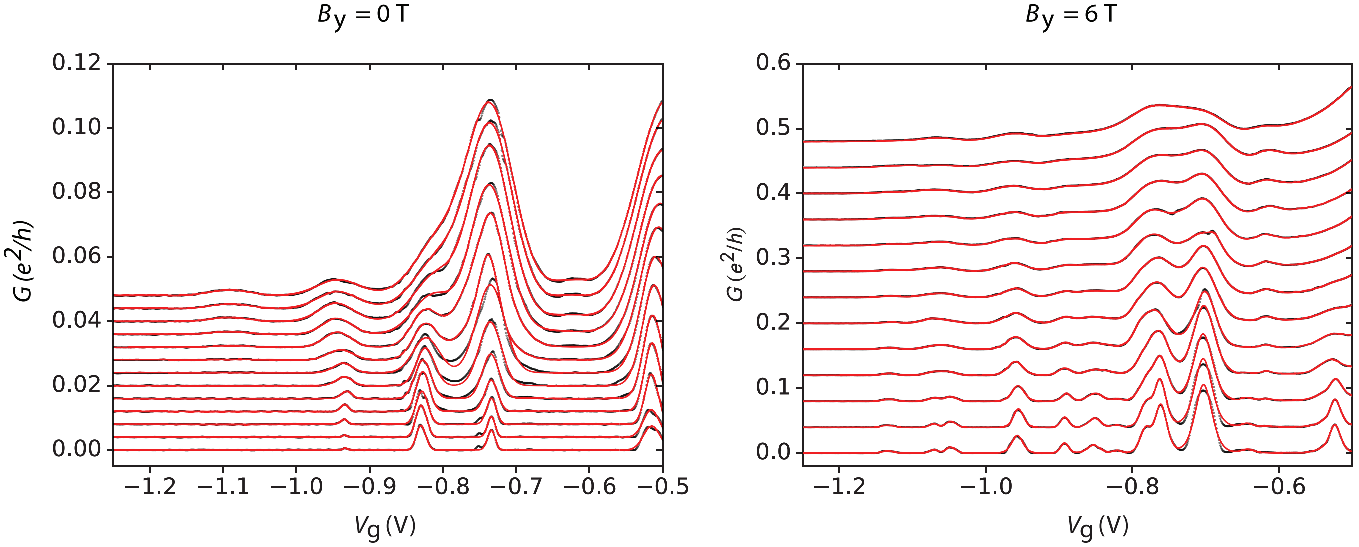

Supplementary figure 11. **Temperature dependence of peak widths and amplitudes.** Temperature dependence of $G(V_\mathrm{g})$ for $B_\mathrm{y} = 0$ T (left panel) and $B_\mathrm{y} = 6$ T (rightmost panel). Traces are taken at varying temperatures and offset vertically. Red lines show the fitted profile to a multi-Gaussian model.

Supplementary Note 6. CHARGE KONDO EFFECT

In the case of $U < 0$, the charge Kondo affect can arise when the tunnel coupling Γ is sufficiently large such that the Kondo temperature $k_\mathrm{B}T_\mathrm{K} = 1/2\sqrt{\Gamma U}\exp[\pi\epsilon_0(\epsilon_0 + U)/\Gamma U] > k_\mathrm{B}T_\mathrm{sample}$. The charge Kondo effect manifests as a higher-order co-tunneling process that enhances conductance at low temperature at the degeneracy points of the even charge states.

In the main manuscript we presented detailed analysis of a single zero bias resonance. Not all resonances in the strong coupling regime show the conductance increase at low temperature. Interpreting the upturn as a consequence of the charge-Kondo model such behavior can be expected as the Kondo temperature depends on the coupling strength which naturally varies between resonances. In Supplementary Fig. 13 we present the corresponding data for a number of additional resonances as indicated in Supplementary Fig. 12. Supplementary Fig. 14 shows the details of the corresponding bias spectroscopy with the widths of the zero-bias resonance extracted both as a function of V_sd and V_g where the latter has been converted to energy using the gate-lever arm estimated from the individual bias spectra. In both cases the widths have been converted in to temperature to allow comparison with the characteristic temperature where the conductance starts increasing upon cooling. The theoretical treatment of the charge Kondo effect in negative-U quantum dots predicts that all three energy scales should be set by the Kondo temperature T_K in reasonable agreement with our findings. We note, however, that the comparison with the theory is complicated by the finite magnetic field $B_\mathrm{z} = 300\,\mathrm{mT}$ which is below the pairing field but required to avoid effect of superconductivity in the leads.

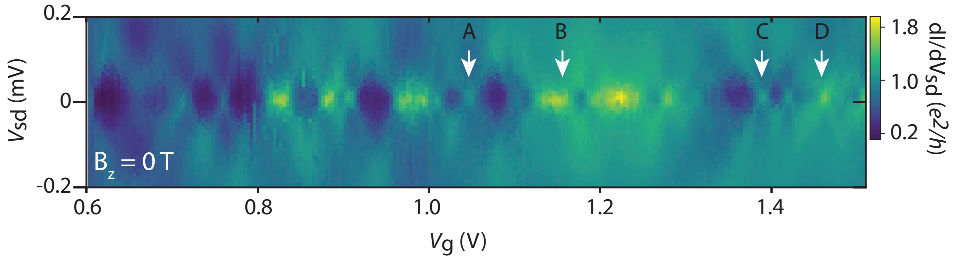

Supplementary figure 12. **Transport spectroscopy overview in strong coupling regime.** Bias spectra in the regime of strong tunnel couplings to the leads. Arrows indicated the resonances for which further data is presented in Supplementary Fig. 13.

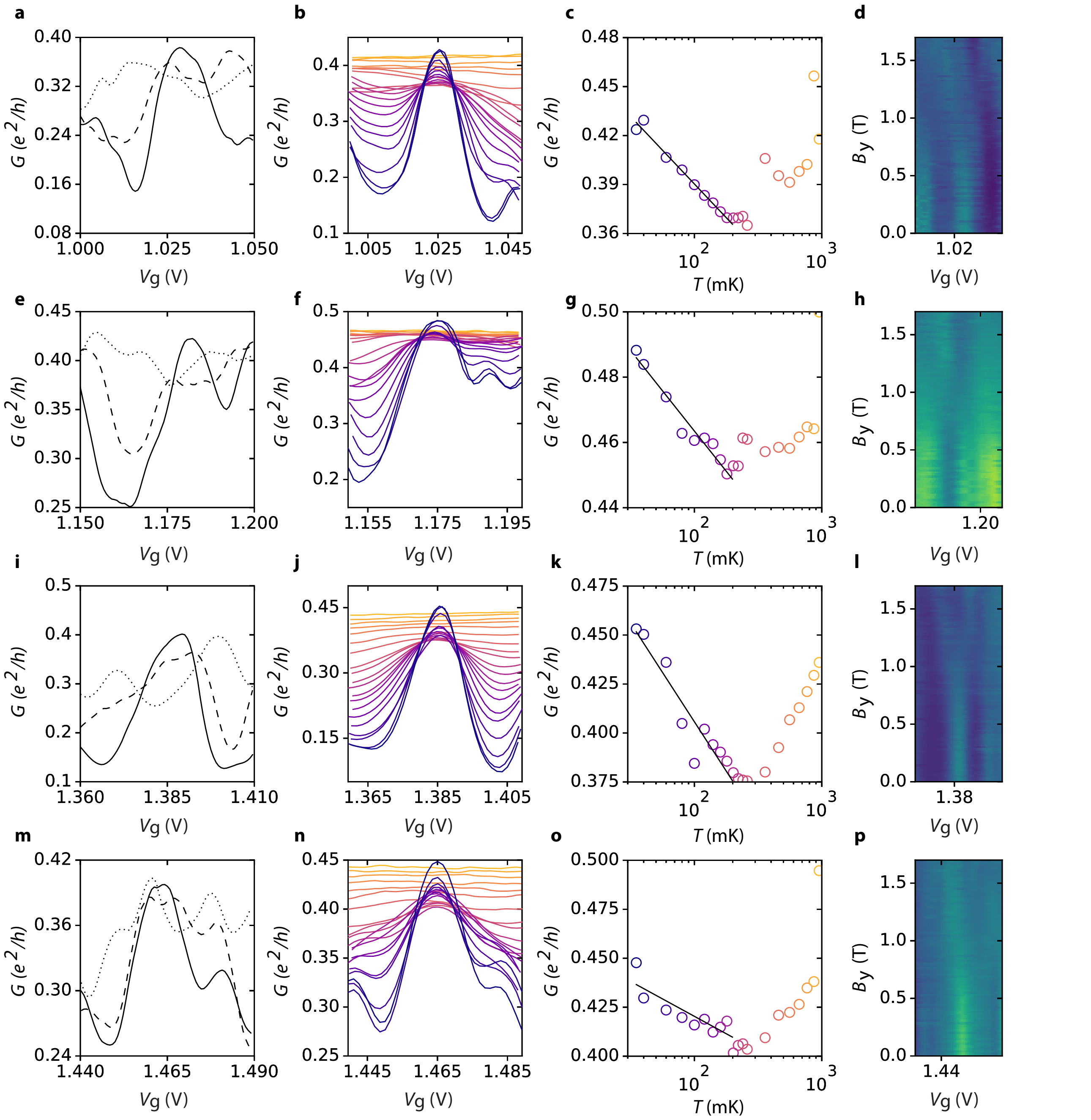

Supplementary figure 13. **Characteristics of resonances in the strong coupling regime** Behavior of resonances (A,B,C,D) labeled in Supplementary Fig. 12. Panels a-d correspond to resonance A, e-h to resonance B, i-l to resonance C and m-p to resonance D. The first column (a, e, i, m) shows gate traces at $V_{\rm sd} = 0\,{\rm V}$ for $B = 0$ (solid lines), $B = 0.7\,{\rm T}$ (dashed lines), and $B = 1.4\,{\rm V}$ (dotted lines). The second column (b, f, j, n) shows temperature dependence with the peak height extracted in third column (c, g, k, o). Column 4 (d, h, l, p) shows the conductance as a function of $V_{\rm g}$ and $B_{\rm y}$

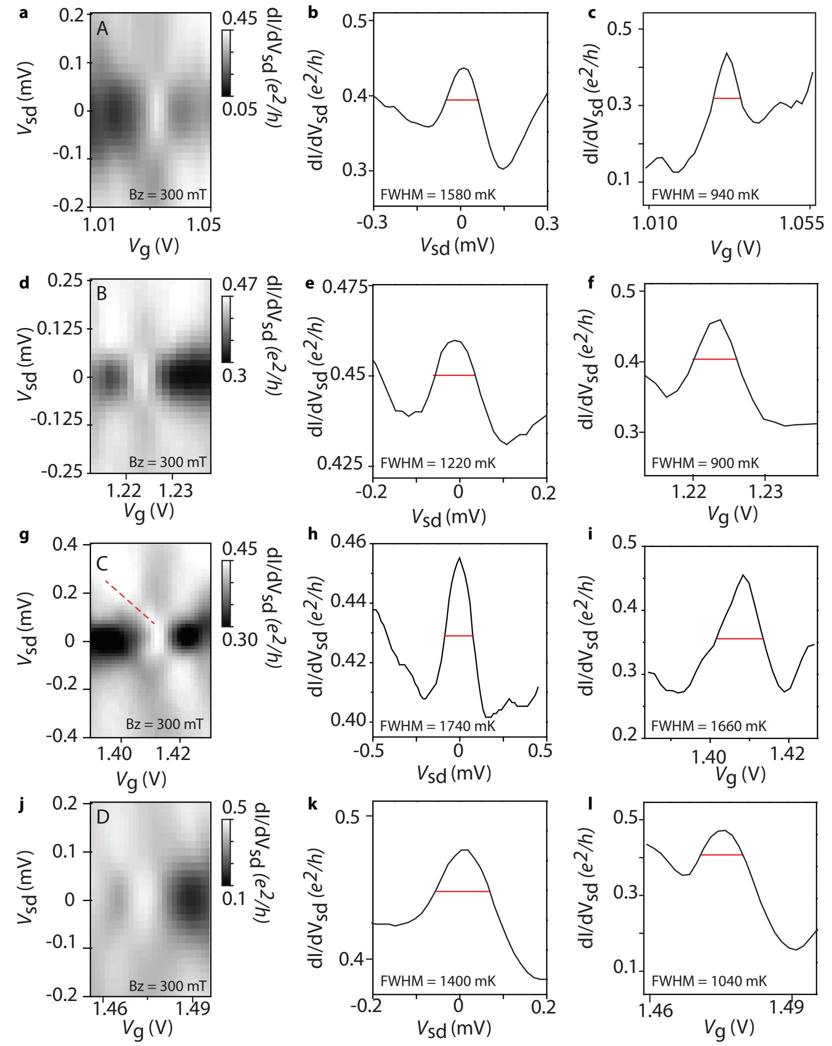

Supplementary figure 14. **Zero-bias peak widths.** Extracted widths of the zero-bias resonances when tuning out of resonance either with the bias (second column: b, e, h, k) or gate (last column: c, f, i, l). The resonances have been labeled according to Supplementary Fig. 12. The characteristic detuning when the conductance has dropped by a factor of two is extracted as indicated after conversion into temperature by Boltzmanns constant. The gate-detuning width was converted into energy using the gate lever-arm determined from the bias spectroscopy in each case (first column: a, d, g, j).

SUPPLEMENTARY REFERENCES

[1] G. E. D. K. Prawiroatmodjo, F. Trier, D. V. Christensen, Y. Chen, N. Pryds, and T. S. Jespersen, "Evidence of weak superconductivity at the room-temperature grown $LaAlO_3/SrTiO_3$ interface," Phys. Rev. B **93**, 184504 (2016).

[2] J. König, H. Schoeller, and G. Schön, "Zero-bias anomalies and boson-assisted tunneling through quantum dots," Phys. Rev. Lett. **76**, 1715–1718 (1996).

[3] M. Leijnse and M. R. Wegewijs, "Kinetic equations for transport through single-molecule transistors," Phys. Rev. B **78**, 235424 (2008).

[4] S. Koller, M. Grifoni, M. Leijnse, and M. R. Wegewijs, "Density-operator approaches to transport through interacting quantum dots: Simplifications in fourth-order perturbation theory," Phys. Rev. B **82**, 235307 (2010).

[5] J. Koch, M. E. Raikh, and F. von Oppen, "Pair tunneling through single molecules," Phys. Rev. Lett. **96**, 056803 (2006).

[6] Thomas Ihn, *Semiconductor Nanostructures: Quantum states and electronic transport* (Oxford University Press, 2010).
